# Local-Level Genetic Diversity and Structure of Matsutake Mushroom (*Tricholoma matsutake*) Populations in Nagano Prefecture, Japan, Revealed by 15 Microsatellite Markers

**DOI:** 10.3390/jof3020023

**Published:** 2017-05-11

**Authors:** Hiroyuki Kurokochi, Shijie Zhang, Yoshie Takeuchi, Engkong Tan, Shuichi Asakawa, Chunlan Lian

**Affiliations:** 1 Asian Natural Environmental Science Center, the University of Tokyo, 1-1-8 Midori-cho, Nishitokyo, Tokyo 188-0002, Japan; zsjtd310@anesc.u-tokyo.ac.jp (S.Z.); lian@anesc.u-tokyo.ac.jp (C.L.); 2 Nagano Prefectural College of Forestry, 4385-1 Shinkai, Kiso, Kiso-gun, Nagano 397-0002, Japan; takeuchi-yoshie@pref.nagano.lg.jp; 3 Graduate School of Agricultural and Life Sciences, the University of Tokyo, 1-1-1 Yayoi, Bunkyo-Ku, Tokyo 113-8657, Japan; tanengkong@gmail.com (E.T.); asakawa@mail.ecc.u-tokyo.ac.jp (S.A.)

**Keywords:** genetic diversity, private allele, microsatellite markers, genet, genetic conservation

## Abstract

The annual yield of matsutake mushrooms (*Tricholoma matsutake*) has consistently decreased in Japan over the past few decades. We used 15 polymorphic and codominant simple sequence repeat (SSR) markers, developed using next-generation sequencing, to carry out genetic analyses of 10 populations in Nagano, Japan. Using the SSRs, we identified 223 genotypes, none of which was observed in more than one population. The mean expected heterozygosity and standardized allelic richness values were 0.67 and 4.05, respectively. Many alleles appeared in only one of the 10 populations; 34 of these private alleles were detected with a mean number per population of 3.4. The fixation index (*F*_ST_) and standardized genetic differentiation (*G*′_ST_) values were 0.019 and 0.028, respectively. Analysis of molecular variance (AMOVA) showed that the contribution of among population, among genets within a population, and within genets variation to the total variation was 2.91%, 11.62%, and 85.47%, respectively, with genetic differentiation being detected for all sources. Twenty-eight of 45 pairwise *F*_ST_ values were significantly larger than zero, and no pattern of isolation by distance was detected among the 10 populations. Bayesian-based clustering did not show clear differences among populations. These results suggest that reestablishment of a colony would be best accomplished by transplantation within a field; if this is not possible, then transplantation from within several dozen kilometers will cause little damage to the original population genetic structure.

## 1. Introduction

In Japan, matsutake mushrooms (*Tricholoma matsutake* (S. Ito et S. Imai) Singer) are a very popular and expensive food item. This mushroom can only be harvested from the wild, since the artificial cultivation of matsutake mushrooms has not yet been successful. The annual yield of matsutake mushrooms has declined steadily in recent decades after peaking in the 1950s [1]. Thus, resource control of *T. matsutake* is an urgent matter.

Management strategies to increase matsutake mushroom yield have been suggested by previous studies and by empirical observation [2]. However, a different approach is required when reintroducing matsutake mushrooms into a field from which they have been lost, since this necessitates the transplantation of individuals from other locations. Optimally, this transplantation would be conducted without disturbing the underlying genetic structure, because indigenous species often contain a spatial genetic structure in their wild populations linked to the specific local ecosystems. Thus, information on genetic diversity, structure, and connectivity is essential to the effective maintenance of matsutake mushroom populations.

Geographic genetic similarity and differentiation of matsutake mushrooms in Asia has been reported at a nationwide scale [3,4,5]. Murata et al. [4] comprehensively assessed genetic differentiation in matsutake mushrooms from Japan and other Asian countries. At a fine scale, the expanding process of matsutake mushrooms has been researched by clarifying the distribution of genets within fairy rings, which are called shiro [6], and new shiro establishment [7]. However, there is almost no information on the genetic structure and connectivity of matsutake mushroom populations at a local level in Japan. Microsatellite (SSR: simple sequence repeat) markers for polymorphism analysis are known to be effective for analyzing the reproductive mechanisms of many organisms. Nuclear SSR markers with high polymorphism might also contribute to the analysis of parenthood, kinship, genetic structure, and population genetics [8,9,10,11,12,13]. Nagano prefecture is one of the most productive areas for matsutake mushroom growth and has recently had the highest annual yield of this product in Japan (Forestry Agency, Ministry of Agriculture, Forestry and Fisheries: Japan Annual report on trends in forest and forestry in Japan, Fiscal year 2009 (Summary)). This study was initiated to gain a better understanding of the genetic structure and genetic connectivity of matsutake mushroom populations at a local level. To this end, we investigated nuclear SSR polymorphisms in populations primarily in Nagano, Japan. Such genetic analyses will provide fundamental information for the development of a successful resource management strategy for matsutake mushrooms.

## 2. Materials and Methods

### 2.1. Sample Collection

**Figure 1:**
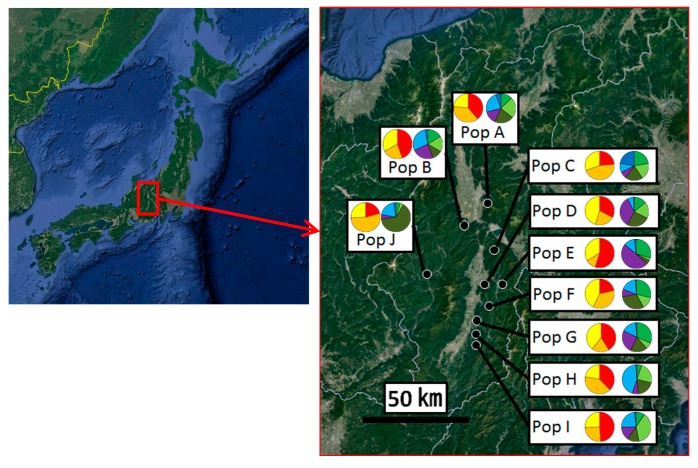
Locations of populations where matsutake mushrooms were collected in Nagano prefecture, Japan (black-filled circles) and the results of Bayesian clustering analyses for *K* = 3 and *K* = 6 (pie charts). The population names correspond with Table 1. The colors of each cluster correspond with Figure 3. Original maps were obtained from Google Earth. Pop: population.

Samples of matsutake mushrooms were collected from 10 different areas in Nagano prefecture, Japan. A population was defined as a sample set collected from the same area; thus, we used 10 populations in this study and the distances between populations were from 3.3 (Populations G–H and H–I) to 53.1 (Populations A–I) km (Figure 1). We asked colleagues, who are owners of each area and have been collecting matsutake mushrooms for more than a dozen years, to collect stipes without worm-eaten from different genets, which they estimated, and to store the stipes separately in bags and keep them frozen until analysis.

### 2.2. Marker Development

New microsatellite markers (simple sequence repeat (SSR) markers) for *T. matsutake* were developed using next-generation sequencing as described by Kurokochi et al. [14,15]. Mushroom stipes without worm-eaten were collected, and pieces of tissue of approximately 125 mm^3^ (5 mm × 5 mm × 5 mm) were obtained. In order to get enough DNA with high quality, genomic DNAs were extracted from these pieces of tissue using a NucleoSpin^®^ Plant II kit (TaKaRa Bio, Kyoto, Japan). Fragmented DNA was produced from the extracted DNA [14,15]. DNA fragments, approximately 200 bp in length, were sequenced using an Ion Proton^TM^ Sequencer (Applied Biosystems, Waltham, MA, USA). The sequences were collected as described by Kurokochi et al. [16] and Sugiura et al. [17]. SSRs were derived using MISA software (http://pgrc.ipk-gate rsleben.de/misa/); 15 regions were selected and Primer3Plus software [18] was used to design primers for these SSRs (Supplementary 1).

### 2.3. Genotyping

In order to extract DNA cheaply from the frozen stipes collected from the different areas, the CTAB method [19] was used. To amplify DNA fragments using the designed markers, an economical variation of the method for amplifying fluorescently-labeled PCR fragments was used [20]; in this method, the fluorescently-labeled primers were replaced by non-labeled primers with a U19 (5′-GGTTTTCCCAGTCACGACG-3′) or M13 (5′-CAGGAAACAGCTATGAC-3′) sequence. PCR was performed in a final volume of 5 µL containing 2× Type-it multiplex PCR master mix (Qiagen, Tokyo, Japan), 0.4 µM reverse primer, 0.1 µM forward primer with a U19 or M13 tail, 0.4 µM fluorescently-labeled U19 or M13, and 5–100 ng genomic DNA. The PCR conditions were as follows: 95 °C for 5 min; 30 cycles of 95 °C for 30 s, 60 °C for 90 s, 72 °C for 30 s; and 60 °C for 30 min. The PCR products were electrophoresed using an ABI 3130 sequencer (Applied Biosystems) and their fragment sizes were detected automatically using GeneMapper software (Applied Biosystems). Genotypes for each sample were then determined from the 15 SSR marker dataset. If the combined exclusion probability of identity (pID) values calculated for two arbitrary samples by CERVUS ver. 3.0.3 [21] were less than 0.001, the two samples were considered to have the same genotype, indicating that they were produced from the same genet.

### 2.4. Statistical Analysis

After removing clonal replicates from each population, the following statistical analyses were conducted. For each population, Nei’s unbiased expected heterozygosity (*H*_E_; [22]), allelic richness (*A*_R_; [23]), and inbreeding coefficients (*F*_IS_) were calculated using FSTAT [24]; this program was also used to test the significance of *F*_IS_ deviations from zero by 1000 random permutations. In addition, the number of private alleles, which were detected from only one population, over markers (*P*_A_) was calculated for each population using GenAlEx version 6 software [25]. Nei’s standard genetic distance with sample size correction (*F*_ST_) and standardized genetic differentiation between populations (*G*′_ST_) were also calculated using FSTAT.

Assuming that the populations belonged to one group, the contributions of among population, among genets within population, and within genets variation to the total variation were calculated by analysis of molecular variation (AMOVA) using the software Arlequin 2.0 [26]. AMOVA was conducted on the dataset reduced to one copy of each genotype. The probabilities of variance for the components were estimated from 1000 random permutations. In addition, genetic differentiation among populations was evaluated using pairwise *F*_ST_ values calculated with Arlequin (1000 random permutations).

Patterns of isolation-by-distance (IBD) were evaluated using a Mantel test between *F*_ST_/(1 − *F*_ST_) (*F*_ST_ values were calculated by FSTAT) and the natural logarithm of geographic distance, using GenAlEx version 6 software [25]. The significance of the Mantel test was estimated using 999 permutations.

Bayesian-based clustering analysis was used for population partitioning with STRUCTURE ver. 2.3.2.1 [27]. After performing 50 independent runs of ln(*K*) for *K* = 1–11 using the admixture model with allelic frequencies correlated among populations and ignoring prior population information, with 50,000 MCMC (Markov chain Monte Carlo methods) repetitions and a burn-in period of 50,000 iterations, the optimal value of *K* was estimated by calculating Δ*K* [28] to identify the top level in the hierarchical structure.

## 3. Results

### 3.1. Genotypes and Genetic Diversity in Matsutake Mushroom Populations

In total, 328 matsutake mushroom samples were analyzed from the 10 populations; 223 genotypes were detected (Table 1) (Supplementary 2). All genotypes grouped within the population from which they were sampled. *H*_E_, *A*_R_, *F*_IS_, and *P*_A_ values for each population are presented in Table 1. The *A*_R_ values are standardized to a sample size of five genotypes per population. The *F*_IS_ values of three populations (Populations F, G, and H) deviated significantly from zero (*p* < 0.05). Private alleles were detected in all populations; in total, 34 private alleles were identified (Table 1). The *F*_ST_ and *G*′_ST_ values were 0.019 and 0.028, respectively.

### 3.2. Genetic Structure and Connectivity

**Figure 2:**
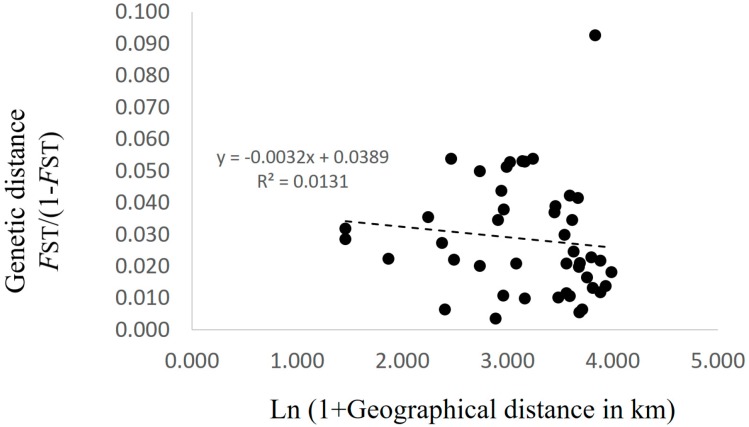
Relationship between the matrix of pairwise differentiation for *F*_ST_/(1 − *F*_ST_) and the matrix of the natural logarithm of geographic distance in the 10 populations in Nagano prefecture, Japan.

AMOVA showed that the contributions of among population, among genets within population, and within genets variation to the total variation were 2.91%, 11.62%, and 85.47%, respectively, with genetic differentiation being detected for all sources (*p* < 0.001; Table 2). Pairwise *F*_ST_ values between populations were calculated for the 223 genotypes. Twenty-eight of 45 values did not differ from zero at a significance level of 0.05 (Table 3). No pattern of IBD was detected among the 10 populations (Figure 2). In the structure analysis, the highest and second highest peaks of Δ*K* were three (Δ*K* = 3.0; clusters 1, 2, and 3) and six (Δ*K* = 2.0; clusters i, ii, iii, iv, v, and vi) (Supplementary 3 and 4).

## 4. Discussion

Matsutake mushrooms (*T. matsutake*) are harvested in several Asian countries, including Japan, China, Korea, and Bhutan [1]. Past studies have demonstrated genetic differentiation of matsutake mushroom populations in these countries; these studies principally compared rDNAs, and only slight genetic variations were reported [3,5,29]. Considering that basidiospore dispersal is geographically limited and geographical distance between a novel genet and a parent genet was at most a dozen meters [7], the existence of genetic differentiation in matsutake mushrooms on an Asian scale is to be expected. Murata et al. [4] demonstrated clear genetic differentiation among matsutake mushrooms harvested in different Asian countries as well as the similarity of those harvested in Japan using retroelement-based DNA markers.

**Figure 3:**
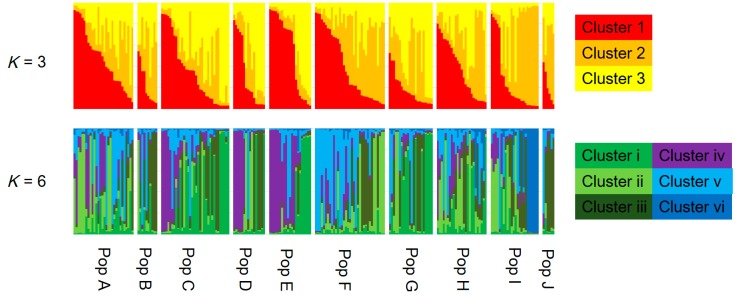
Assignment of each genotype (vertical bar) to genetic clusters 1, 2, and 3 in *K* =3, and clusters i, ii, iii, iv, v, and vi in *K* = 6 using a model-based Bayesian clustering method (STRUCTURE ver. 2.3.2.1). The population names correspond with those presented in Table 1.

Xu et al. [30] reported a significant positive correlation between genetic distance and geographical distance among matsutake populations in southwestern China. They used 17 populations with two to 19 samples per population, and the geographic distance between each pair of populations ranged from a dozen to more than 1000 km [30]. In contrast, Lian et al. [6] reported no genetic differentiation among six populations in Iwate, Japan that were within 500 m of each other. In this study, the geographic distances among the 10 populations in Nagano prefecture ranged from 3.3 to 53.1 km, and no significant IBD was detected (Figure 2). Moreover, the Bayesian-based clustering analysis did not identify clear differences among populations (Figure 3). In addition, the standardized genetic values were not large, with *F*_ST_ = 0.019 and *G*′_ST_ = 0.028. These results suggested that genets of *T. matsutake* are established by the long-distance dispersal of basidiospores. Although Narimatsu et al. [7] reported that geographical distance between a novel genet and a parent genet was at most a dozen meters at a fine-scale study within 500 m, our results did not deny the possibility that basidiospores of *T. matsutake* could travel for a longer distance at the scale of this study. Thus, in the prefecture scale in Japan, i.e., at the local scale, genetic distance might not be dependent on geographic distance. To determine the existence of increased genetic difference beyond prefecture boundaries, it will be necessary to investigate populations from different prefectures in the future.

The indexes we used to describe genetic diversity (*H*_E_, *A*_R_, and *F*_IS_) were in the same range in all 10 populations (Table 1). As our colleagues in Nagano prefecture collected consistent numbers of matsutake mushrooms annually from the fields where our samples were obtained, it is possible that these levels of genetic diversity represent the range required to maintain matsutake mushroom yields in Nagano.

Although there was no significant IBD in this study (Figure 2), some pairwise *F*_ST_ values were significantly larger than zero (Table 3) and AMOVA showed significant genetic differentiation among the populations (Table 2). In addition, several clusters obtained by Bayesian-based clustering methods centered on only a few populations; for example, cluster iv was mainly from Populations C, D, and E, while cluster vi was mainly from Population I (Figure 3). Interestingly, cluster vi was clearly genetically distant (Supplementary 4), indicating that those matsutake mushrooms derived from cluster vi might have high local endemism. Our results demonstrated that genetic differentiation could be detected among populations of matsutake mushrooms even within 3.3 km (Populations H–I) of each other (Table 3). In addition, private alleles were detected from all populations in Nagano. These results indicate that each population might be derived from different colonizing events or have a long history of establishment in each location.

If the considerable efforts of related research organizations lead to the establishment of transplantation methods for matsutake mushrooms, it will be important to conserve their genetic resources at a local level. The present study, which presents an example of genetic diversity at the local level in Nagano prefecture, demonstrates the genetic similarities and differences that need to be taken into account. Since each matsutake population includes its own genetic resources and history (Table 2 and Table 3), as indicated by the prevalence of private alleles (Table 1), individuals should be transplanted, if possible, from within the same field. However, if necessitated by the loss of all specimens and spores of matsutake mushrooms from a field, our results from Nagano prefecture suggest that transplantation from up to several dozen kilometers away will cause little damage to the original genetic structure, since significant IBD was not detected (Figure 2) and the clustering with STRUCTURE ver. 2.3.2.1 did not show the clear differences among populations (Figure 3) with the low values of *F*_ST_ and *G′*_ST_. In addition, for the sustainable harvesting of matsutake mushrooms, genetic diversity as described by *H*_E_, *A*_R_, and *F*_IS_ (Table 1) could be a possible indicator to assess transplanted fields.

Considering these results, the reestablishment of a colony would be best accomplished by transplantation within a field; if this is not possible, then transplantation from within several dozen kilometers will cause little damage to the original population genetic structure.

## Figures and Tables

**Table 1:** Baseline parameters of the 10 populations of matsutake mushrooms in Nagano prefecture, Japan, including sample sites, baseline data, and genetic diversity based on 15 nuclear microsatellite markers.

Population	Longitude	Latitude	N	G	*H*_E_	*A*_R_	*P*_A_	*F*_IS_
Pop A	138°02′	36°11′	42	30	0.68	4.30	4	0.090
Pop B	137°54′	36°05′	21	10	0.66	4.09	2	−0.032
Pop C	138°04′	35°58′	41	34	0.70	4.11	3	0.090
Pop D	138°00′	35°49′	25	16	0.60	3.67	4	0.133
Pop E	138°05′	35°45′	37	21	0.66	3.74	1	0.119
Pop F	138°01′	35°42′	43	35	0.68	4.21	6	0.086 *
Pop G	137°57′	35°37′	38	22	0.67	4.03	6	0.142 *
Pop H	137°58′	35°35′	33	25	0.67	4.05	2	0.165 *
Pop I	137°57′	35°33′	29	24	0.69	4.18	4	0.066
Pop J	137°41′	35°51′	19	6	0.65	4.14	2	−0.006
			(Total)		(Average)			
			328	223	0.67	4.05	3.4	0.085

Note: N: number of samples analyzed, G: number of detected genotypes, *H*_E_: expected heterozygosity, *A*_R_: allelic richness (*n* = 5), *P*_A_: number of private alleles, *F*_IS_: inbreeding coefficient. Asterisks (*) indicate that the value of *F*_IS_ is significantly higher than zero.

**Table 2:** Results of analysis of molecular variance (AMOVA) on the contribution of each source to total variation.

Source of Variation	D.F.	Sum of Squares	Variance Components	Percentage of Variation
Among populations	9	44.5	0.06 *	2.91
Among genets within populations	213	484.7	0.24 *	11.62
Within genets	233	399.0	1.79 *	85.47
Total	445	928.2	2.09	100

D.F.: degree of freedom, Asterisks (*): *p* < 0.001.

**Table 3:** Pairwise *F*_ST_ values between 10 populations of matsutake mushrooms.

Population	Pop A	Pop B	Pop C	Pop D	Pop E	Pop F	Pop G	Pop H	Pop I	Pop J
Pop A		−0.003	0.012	**0.055**	**0.045**	0.003	0.001	0.015	**0.020**	**0.037**
Pop B			**0.040**	**0.088**	0.032	0.017	0.008	0.019	0.027	**0.074**
Pop C				**0.058**	**0.060**	**0.015**	0.009	**0.036**	**0.020**	0.023
Pop D					**0.085**	**0.048**	**0.048**	**0.070**	**0.097**	**0.095**
Pop E						**0.043**	**0.053**	**0.029**	**0.061**	**0.127**
Pop F							0.000	**0.025**	**0.033**	0.036
Pop G								0.019	0.007	0.029
Pop H									**0.038**	**0.081**
Pop I										**0.040**
Pop J										

Bold: values significantly higher than 0 (α = 0.05).

## Supplementary Materials

Supplementary materials can be found at www.mdpi.com/2309-608X/3/2/23/s1.
