# Supplementary material for: Local-Level Genetic Diversity and Structure of Matsutake Mushroom (Tricholoma matsutake) Populations in Nagano Prefecture, Japan, Revealed by 15 Microsatellite Markers
Source: J Fungi (Basel). 2017 May 11;3(2):23. doi: 10.3390/jof3020023 (PMC5715919; doi:10.3390/jof3020023)
Supplement: Supplementary file 1 [file jof-03-00023-s001.zip › Supplementary 1.docx]

**Supplementary**

**Amplification primers and characteristics of 15 polymorphic microsatellite regions in *T. matsutake***

| Locus ID | Primer sequence (5'-3') | | Motif repeat |
| --- | --- | --- | --- |
|  | Forward | Reverse |  |
| TM-001 | (U19)-TGCCCTCATTCAATTCTTCC | GTGCTTGGCGCTAGAAAAAC | (TC)13 |
| TM-002 | (U19)-CGTCTTTCTTGTCCCACACA | CACATCATGGACAATGCACA | (AG)18 |
| TM-003 | (U19)-GGTGATGATGACCACATGGA | GTGCAGCAGGACAGAAGACA | (GAT)12 |
| TM-004 | (M13)-CCTGACCAGGCACAGTAGAA | GAACCTTCAGAACCCAGCAT | (AGT)11 |
| TM-005 | (U19)-TGGAGCCACAATGACAGAAA | CACCACTGTTTTGGTTGTGC | (ACA)12 |
| TM-006 | (M13)-GATAGCGGGCGTACTCAAGA | GCTTGTCGACAGCAATGAAA | (AAC)10 |
| TM-007 | (M13)-TGTGGTTTTGGATGGTTGAA | GGCAATTTTTGAACCCCAAT | (GTT)10 |
| TM-008 | (U19)-GGGGGATTCAATTGAGGTTT | ATGTCCTTTCCCCCTTGTTC | (GTT)10 |
| TM-009 | (M13)-GATGACACAGGCAACACTCG | ATCAGGAATTGAGCCTGTCG | (CAA)12 |
| TM-010 | (U19)-GCCGCTTTAAACGAGCTCTA | CCACGTGTTCGTTTCTTTGA | (TTTC)8 |
| TM-011 | (M13)-CAAGAACCCTCCTTCGTCTG | GGGTCGGACTCCTTCTTTTC | (AGAGAC)7 |
| TM-012 | (U19)-TGTCCTAGATGTGGGGGAAG | ATGAATTCAGCTCGGACTCG | (CGTGTT)7 |
| TM-013 | (M13)-ACCGTGACAGGTCTCGTAGC | GGGTCGGACTCCTTCTTTTC | (AGAGAC)7 |
| TM-014 | (U19)-GCCTTTTTCGGGTTCAAGTT | ACCATCCACCAAACCGTAAA | (TGATGGACG)4 |
| TM-015 | (U19)-CCACTTTTGGTCGTGGAAGT | CACCTGTAGCACCATCGAGA | (CTGGCAGTGGAAGTC)4 |

U19 and M13 in the primer sequence indicate a tail of ‘‘GGTTTTCCCAGTCACGACG’’ and ‘‘CAGGAAACAGCTATGAC’’, respectively.

**Sequences of each regions amplified by the developed primers in this study**

TM-001

TGCCCTCATTCAATTCTTCCTCATTTCTCTCTCTCTCTCTCTCTCTCTCTCTAGACCCGAAAACATCCTCTATCGAACGAAAGACCCCGGAAGTGACATTGTGATTGCGGATTTCGGAATGTCCGTCGAGAGTCTCCTCAAGCTGCCTCGCTAATGTCCAAGGTTTTTCTAGCGCCAAGCAC

TM-002

CGTCTTTCTTGTCCCACACAAAAGATAGAAAACACACATAGAGAGAGAGAGAGAGAGAGAGAGAGAGAGAGAGAGCAGAGCAGATAAATCAATAGACGTCACGCAGCGCATTTATTTTGAAAAATGAAGAGTTAGTTATTATGAGCAGAGCTCAGGATCCGGTGTGCAAAACATCACGGAAAGTCCTCCCGTCGAGGAAGGCTGGACATGCGGGGGGGCTGTATGTGCATTGTCCATGATGTG

TM-003

GGTGATGATGACCACATGGAGGATGAGGACAGGGATGATGATGATGATGATGATGATGATGATGATGATGAAGGCAACCACCTGCAGAAAGAGGTAAAAAGGTACTGCAAAAAGGCTGGGGGTGACAAATGTGATGCTGAAGATGGCCCAGACTGGATGTTTGATGCTGAAGAAGTTACCTCAAAGGATCCTTCCTATGTCTTCTGTCCTGCTGCAC

TM-004

CCTGACCAGGCACAGTAGAACTTGGGAACTTGAGTCTTATCCAAAAATATCACACCAAAGTTTATCTGGATTGGACCAATCTTATCCAAGAACAGATGAAAATGGATTTATGGGGTCCTTATGTTAATCATCAACCAGTAGTAGTAGTAGTAGTAGTAGTAGTAGTAGTAAATACATGGCATGGTCCCTAATAAGAAATGCTGGGTTCTGAAGGTTC

TM-005

TGGAGCCACAATGACAGAAACAATGGGATTGGATGACACCGACACCAATAATGAGGGGATAGACAACAACAACAACAACAACAACAACAACAACAACAGGGACCAGGGGATGAAGACCAACAATGAGGAATATGATGACAAGGACAACCACGAGACAATGATGGACAACAAGGACAACCACCACAAGACAATGACAGGGCCAGAGACAGAGATGGACAACAACACTGCACAACCAAAACAGTGGTG

TM-006

GATAGCGGGCGTACTCAAGATGAGATAATTTAGCAGAAAACAAAGTCCACCTGGATGGACCCCGAAAGAAATCGCATCTCATCCCAATTGAACAACAACAACAACAACAACAACAACAACCATCACCACCCACTGCTCGTGCGGGATTGGGCCTTGGTTGCTTGCTGGTTGCTCGTTCCGGCCGCTACGAGGACAGGACTTTCATTGCTGTCGACAAGC

TM-007

TGTGGTTTTGGATGGTTGAAAGTTCTTTGTAGGTTAAGCAAGATTGACAAGAGTATTATGTAGTGGATTTAGAGGGACATGGTCACAGATAGTGACTTTTTAGAGATGGGGTGGGGTGGGGTTGTTGTTGTTGTTGTTGTTGTTGTTGTTGCACTGCCTCATGTGTGGAATGATCATCTTTGAGATAGATAACTCCTCAAAAATTGGGATTATTGGGGTTCAAAAATTGCC

TM-008

GGGGGATTCAATTGAGGTTTGAGGATGACTTTCTATTGATTTGGCACCAAGAAACTAAAGGATTCTTTTTTTTGGTTTTGGGCACTGGACTGGTTGTTGTTGTTGTTGTTGTTGTTGTTGTTAGGCTGGGTACAGTGTACACAGGTACAGGTGTGGTGTGTGAAATTTACTCATGGTGTGACCCATGCTACACCCTAGGGATGATGGGGATGCAGGGGAACAAGGGGGAAAGGACAT

TM-009

GATGACACAGGCAACACTCGTAAAGCTAGGCGGCTGTACTGTATAAGATATTCTCATGTTCTCAATATGCTGCTTGTCATCAGATCAATAAGCCCATCCAACAACAACAACAACAACAACAACAACAACAACAACCACTGCCCACCGCTCGTGCGGGATAGGGCCCTGGTTGCTTGCTGGTTGCTCGTTCCGGCCGCTATGAAGACAGGACTTTCGTTGCCGTTCGACAGGCTCAATTCCTGAT

TM-010

GCCGCTTTAAACGAGCTCTAATAAAAGATGAACACCCCCTCTTTAGACCTTCGCACTTCTTTGGCTGCGAGGCTTGGCATGTATGTTTTCTTTCTTTCTTTCTTTCTTTCTTTCTTTCCATATCATATCTAATCTATATGTCAAGGGAGACAAAGACTGTAAATGCTTGGTTTCAGAATAAAAGAGCATCCTCAAAGAAACGAACACGTGG

TM-011

CAAGAACCCTCCTTCGTCTGTCTGGCGCTATGATAGGCGTGTAATCCCCACCCCACAGGTCGTCCCGTAGCGATCGCGACCGTGACAGGTCTCGTAGCCTCGCCGCCGCCCCGAAAGAGACGATCGTGATAGAGACAGAGACAGAGACAGAGACAGAGACAGAGACAGAGACCTCGATAGGGATCGAGAGAGGAGAAGGAGAAGCAGAACCCCGGAGAAAAGAAGGAGTCCGACCC

TM-012

TGTCCTAGATGTGGGGGAAGAGAGGCGTGTTCGTGTTCGTGTTCGTGTTCGTGTTCGTGTTCGTGTTCGTGGTCATGGTCATGCGGGTTGACGACTAATGCGTGATGTAGTGATACAGGATTGGTTGGTTTATATGCTTTATTATGCTGATTCGAGTCCGAGCTGAATTCAT

TM-013

ACCGTGACAGGTCTCGTAGCCCTCGCCGCCGCCCCGAAAGAGACGATCGTGATAGAGACAGAGACAGAGACAGAGACAGAGACAGAGACAGAGACCTCGATAGGGATCGAGAGAGGAGAAGGAGAAGCAGAACCCCGGAGAAAAGAAGGAGTCCGACCC

TM-014

GCCTTTTTCGGGTTCAAGTTCAAAATTTAAGTCTGAAGTGAGGGTGAATTGATGGACGTGATGGACGTGATGGACGTGATGGACGTGCTTTCTGTCGGCACCTCGGCGAGGACCCTGAACGCCACCGATTTGGAGTCAAGTTAGCGTTACGGAAAGTCAATAGTTCCTATTACTAAATAAGTTATTCCTGTAATAAAATTGGTAATTTACGGTTTGGTGGATGGT

TM-015

CCACTTTTGGTCGTGGAAGTCTTGGCGCTGTTGCTGCAAGTGGAAGTCTATCTGGTGGTGGAACTCCTGGTGGCAGCGCCACTGGCAGTGGAAGTCCTGGCAGTGGAAGTCCTGGCAGTGGAAGTCCTGGCAGTGGAAGTCCCGGTGGTGCTTCTGATAGAAGCTCTGGTGGTGGCGCTACTGGTGGTAGAAGTTCCGCTGGTGGAGGTCCTGGCAGCGCTGACCATCTCGATGGTGCTACAGGTG
