# Supplementary figures and images for: Local-Level Genetic Diversity and Structure of Matsutake Mushroom (Tricholoma matsutake) Populations in Nagano Prefecture, Japan, Revealed by 15 Microsatellite Markers
Source: J Fungi (Basel). 2017 May 11;3(2):23. doi: 10.3390/jof3020023 (PMC5715919; doi:10.3390/jof3020023)

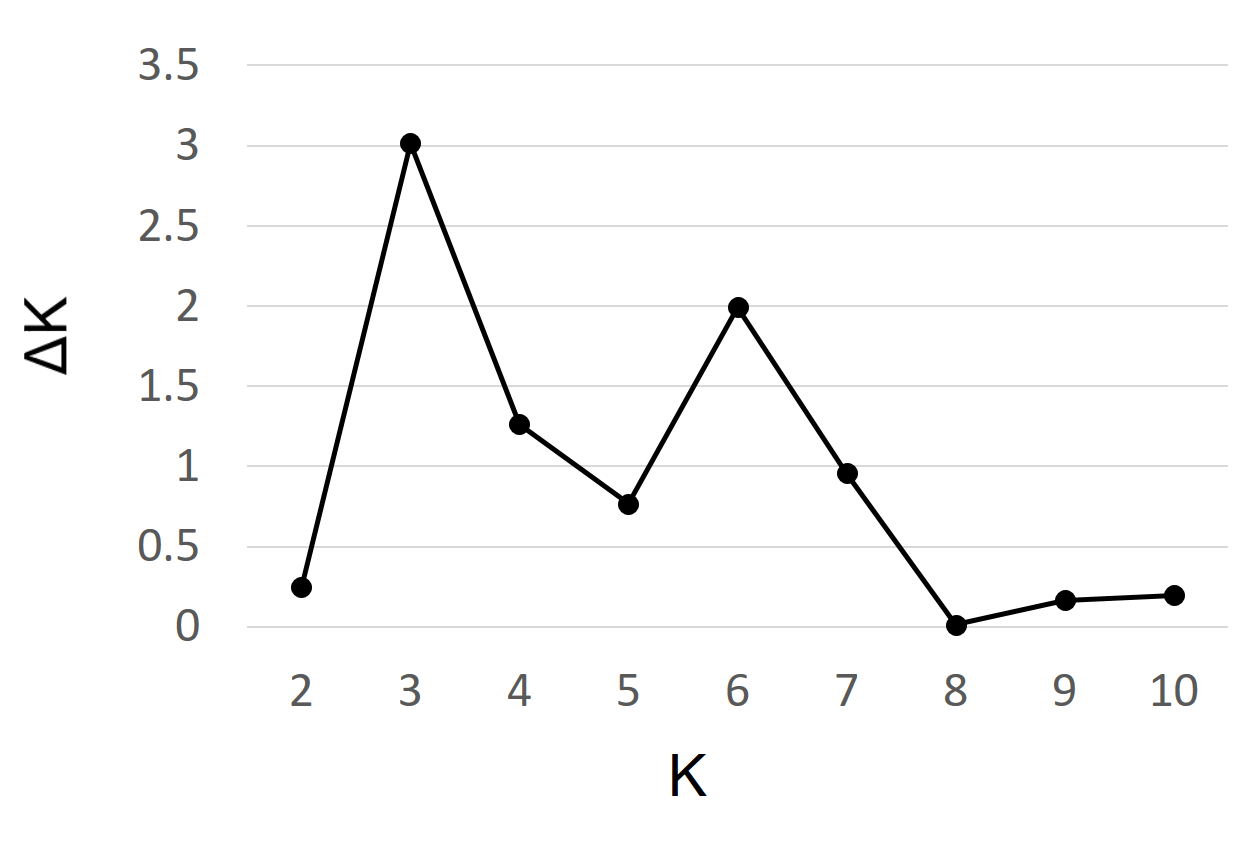

Supplement: Supplementary file 1 [file jof-03-00023-s001.zip › Supplementary 3.bmp]

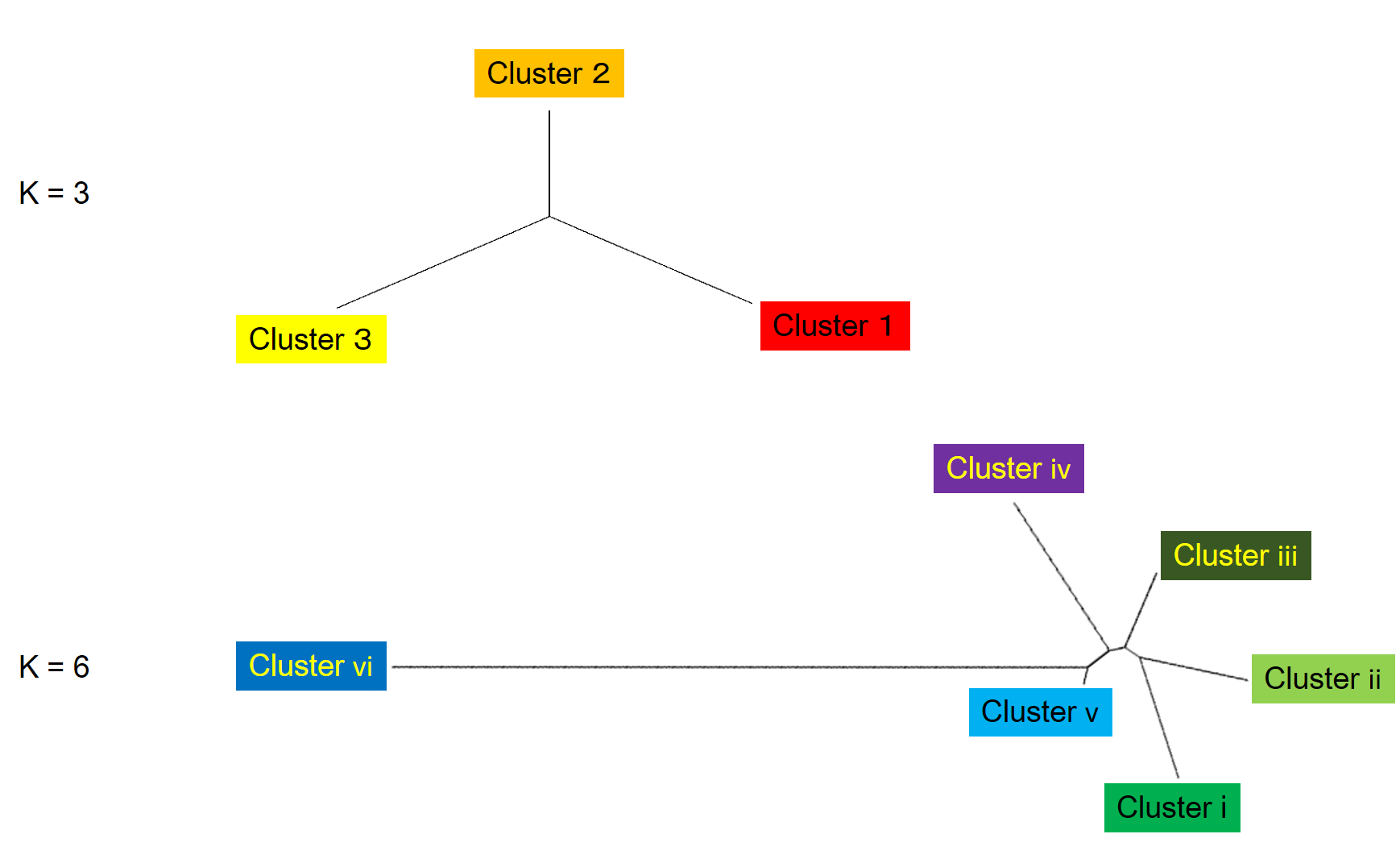

Supplement: Supplementary file 1 [file jof-03-00023-s001.zip › Supplementary 4.bmp]
